# Individual Epitope-Specific CD8^+^ T Cell Immune Responses Are Shaped Differently during Chronic Viral Infection

**DOI:** 10.3390/pathogens12050716

**Published:** 2023-05-14

**Authors:** Sebastian Klein, Jasmin Mischke, Finn Beruldsen, Immo Prinz, Dinler A. Antunes, Markus Cornberg, Anke R. M. Kraft

**Affiliations:** 1 Department of Gastroenterology, Hepatology and Endocrinology, Hannover Medical School, 30625 Hannover, Germanycornberg.markus@mh-hannover.de (M.C.); 2 Twincore Centre for Experimental and Clinical Infection Medicine, 30625 Hannover, Germany; 3 Center for Nuclear Receptors and Cell Signaling, Department of Biology and Biochemistry, University of Houston, Houston, TX 77204, USA; 4 Institute of Systems Immunology, University Medical Center Eppendorf, 20251 Hamburg, Germany; 5 Institute of Immunology, Hannover Medical School, 30625 Hannover, Germany; 6 German Centre for Infection Research (DZIF), 30625 Hannover, Germany; 7 Centre for Individualised Infection Medicine (CIIM), c/o CRC Hannover, 30625 Hannover, Germany

**Keywords:** CD8^+^ T cells, T cell receptor repertoire, chronic viral infection, checkpoint inhibitor therapy, lymphocytic choriomeningitis virus

## Abstract

A hallmark in chronic viral infections are exhausted antigen-specific CD8^+^ T cell responses and the inability of the immune system to eliminate the virus. Currently, there is limited information on the variability of epitope-specific T cell exhaustion within one immune response and the relevance to the T cell receptor (TCR) repertoire. The aim of this study was a comprehensive analysis and comparison of three lymphocytic choriomeningitis virus (LCMV) epitope-specific CD8^+^ T cell responses (NP396, GP33 and NP205) in a chronic setting with immune intervention, e.g., immune checkpoint inhibitor (ICI) therapy, in regard to the TCR repertoire. These responses, though measured within the same mice, were individual and independent from each other. The massively exhausted NP396-specific CD8^+^ T cells revealed a significantly reduced TCR repertoire diversity, whereas less-exhausted GP33-specific CD8^+^ T cell responses were rather unaffected by chronicity in regard to their TCR repertoire diversity. NP205-specific CD8^+^ T cell responses showed a very special TCR repertoire with a prominent public motif of TCR clonotypes that was present in all NP205-specific responses, which separated this from NP396- and GP33-specific responses. Additionally, we showed that TCR repertoire shifts induced by ICI therapy are heterogeneous on the epitope level, by revealing profound effects in NP396-, less severe and opposed effects in NP205-, and minor effects in GP33-specific responses. Overall, our data revealed individual epitope-specific responses within one viral response that are differently affected by exhaustion and ICI therapy. These individual shapings of epitope-specific T cell responses and their TCR repertoires in an LCMV mouse model indicates important implications for focusing on epitope-specific responses in future evaluations for therapeutic approaches, e.g., for chronic hepatitis virus infections in humans.

## 1. Introduction

Chronic viral infections such as hepatitis B virus (HBV), hepatitis C virus (HCV) or human immunodeficiency virus (HIV) are a global health problem with high morbidity and mortality [1]. CD8^+^ T cells are one of the most important determinants for infection progression and can range from highly effective to transient and non-functional. A main reason for the latter is that in chronic viral infection, virus-specific T cell responses are exhausted [2,3], which is associated with suppressive signaling pathways in CD8^+^ T cells, so-called checkpoint pathways [4,5,6].

While the exhaustion of a T cell response was long seen as a linear process that is highly dependent on viral load and time [7,8,9,10], focused research has revealed more diverse effects, such as epitope-specific differences within a single immune response [11,12]. The great advantage of animal models such as the lymphocytic choriomeningitis virus (LCMV) mouse model is the analysis of different parameters under standardized circumstances, which has revealed that the epitope-specific immune responses can be seen as individually distinct parts of the overall “immune response” [12]. During the acute phase, some epitopes induce strong immune responses from the host, while others are less immunogenic. This has been shown in mice [12] and humans [13]. Additionally, some knowledge has been gained about the phenotypical composition and functionality of T cell responses, also in chronic viral infections [14,15,16]. These have shown the massive variance that can occur within one immune response, originating from the different individual epitope-specific responses.

In recent years, markers were defined to characterize different T cell phenotypes in chronic viral infections (e.g., stem-cell-like, terminally exhausted T cells) [15,17,18,19,20]. In this regard, it became clear that some of these T cell phenotypes are potentially restorable and therefore drug targets [21,22]. These drugs, called immune checkpoint inhibitors, block suppressive checkpoint pathways. Predominantly, the blockage of the PD-1/PD-L1 and CTLA-4 pathways received much attention for being able to restore CD8^+^ T cell responses from sensitive T cell phenotypes [13,23,24,25].

Although immune checkpoint inhibitor (ICI) therapy (e.g., αPD-L1 or αCTLA-4 therapy) is already clinically used in cancer treatment [26], the long-term impact of this therapy on the T cell response is only marginally understood.

While the phenotype of T cells, which are sensitive for restoration, has been intensively investigated [21,22,27,28,29,30], the T cell receptor (TCR) repertoire and the effects of ICI therapy have hardly been studied [31]. The TCR is an essential feature of each CD8^+^ T cell and controls the predominant activation of these cells (TCR-dependent activation) by binding to a specific, presented epitope [32]. These TCR–peptide–MHC interactions are crucial for the development of the antigen-specific T cell [33], determining its phenotype [34,35,36], the strength of its proliferation [37] and its functionality [38], which, in turn, influence the outcome of the infection [39]. Current data on epitope-specific TCR responses are sparse and mainly based on single-epitope-specific TCR experiments, such as the transgenic P14 T cells responsive to GP33 [40]. Additionally, the comparison of different organs or the phenotypical subgroup analysis of certain responses has been conducted [41].

Usually, many TCRs bind to a given presented epitope (respective T cells are called “precursor cells” for this epitope) [42], building the responsive epitope-specific TCR repertoire. The diversity of a TCR repertoire in response to a virus has been proven critical for viral persistence or control [43,44,45]. We have recently shown in a chronic LCMV mouse model that exhaustion-dependent loss of the NP396-specific CD8^+^ T cell response is accompanied by a significant reduction in the CD8^+^ T cell clonotype number and diversity index [46]. Only a part of those exhausted T cells seemed sensitive to αPD-L1-treatment-dependent restoration and induced a significant skewing towards oligoclonality with the appearance of dominant, hyperexpanded (more than 25% of relative abundance) NP396-specific clonotypes in ICI-treated mice [46]. In our current work, we focused on the response against the three LCMV epitopes, NP396, GP33 and NP205 [42], and performed a direct comparison within single mice, which, to our knowledge, has not been studied previously [47]. Thereby, we took advantage of the LCMV model, which enables us to analyze epitope-specific immune responses under acute-resolving (LCMV Armstrong, LCMVarm, further termed “immune”) as well as chronic (LCMV Armstrong clone 13, LCMVcl13, further termed “chronic”) conditions [48,49].

Considering that NP396-, GP33- and NP205-specific T cell responses show different phenotypes during chronic infection [12], we asked whether this also results in differentially affected TCR repertoires compared with LCMV-immune mice and how these epitope-specific repertoires are modulated by ICI therapy.

## 2. Materials and Methods

### 2.1. Ethics Statement

For all animal experiments, the highest possible ethical standards were ensured, and all efforts were made to reduce the suffering of mice. All mouse experiments were performed in accordance with the guidelines of the Medical School Hannover (MHH), Germany, the national animal protection law and the animal experiment regulations. The study was approved by the State of Lower Saxony (LAVES—Niedersächsisches Landesamt für Verbraucherschutz und Lebensmittel-sicherheit—project number 33.12–42502-04-16/2127).

### 2.2. Mice and Treatment

Male C57BL/6 mice, 6 to 8 weeks old, were bred and kept under pathogen-free conditions, with a 12/12 h day/night cycle, in the general animal facility of MHH. Mice were infected with 5 × 10^4^ PFU LCMV Armstrong, and were considered immune 4 weeks post infection or were infected intravenously with 2 × 10^6^ PFU of LCMVcl13 to generate chronically LCMV-infected mice. In contrast to some published studies, we did not use a deep-exhaustion LCMV mouse model, in which CD4^+^ T cells are depleted prior to LCMVcl13 infection, which results in a deeper and more stable, but also in a more non-physiological, chronic LCMV infection [12,29,48]. Checkpoint inhibitor treatment using αPD-L1 started at day 23 post LCMVcl13 infection. Mice were treated five times (every third day) intraperitoneally with 200 μg of αPD-L1 (10F.9G2 [BioXcell and Biolegend]), while as control, chronic LCMVcl13-infected mice were treated with phosphate-buffered saline. On day 36 post infection, mice were sacrificed and immune responses and viral titer were analyzed. NP396 data, related to dextramer staining, IFN-γ- and TNF-α-response and TCR sequencing, was in part (~40%) published already in Klein et al. (2020) [46], but was extended (more than doubled) and reanalyzed for this comparative study.

### 2.3. Synthetic Peptides and Dextramers

Synthetic peptides NP396-404 (FQPQNGQFI), GP33-41 (KAVYNFATC) and NP205-212 (YTVKYPNL) were purchased from ProImmune (Oxford, UK). The dextramers H-2D^b^/FQPQNGQFI (NP396), H-2D^b^/KAVYNFATC (GP33) and H-2K^b^/YTVKYPNL (NP205) were purchased from Immudex (Koppenhagen, Denmark).

### 2.4. Cell Surface and Dextramer Staining for Flow Cytometry and Intracellular Cytokine Measurements

**Figure A1:**
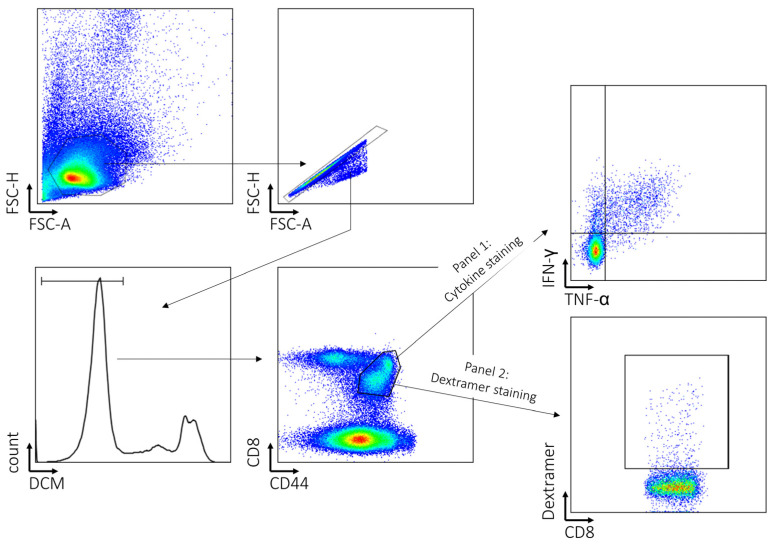
Flow cytometric gating strategy for IFN-γ^+^ and Dextramer^+^ T cells.

All methods were described previously [46,50]. Briefly, red blood cells were removed from single-cell spleenocyte suspensions. Afterwards, ≥1 × 10^6^ cells were either directly stained or stimulated with one of the three peptides within RPMI medium (Gibco) for 4.5  h at 37 °C and 5% CO_2_. After incubation, surface and intracellular stainings were performed (as described previously [46] with GolgiPlug (BD)). Flow cytometry was performed with a BD LSRFortessa flow cytometer (BD; 3 lasers and 14 colors) and data were analyzed with software FlowJo 9 and 10 (BD). Experimental gating strategy is shown in Figure A1.

### 2.5. Next-Generation Sequencing of the Epitope-Specific T Cell Receptor Repertoire

TCR repertoire analyses were performed on epitope-specific CD8^+^ T cells, which were defined by fluorescence-activated cell sorting (FACS), using the CD4^−^ CD8α^+^ CD44^+^ dextramer-binding (Dextramer^+^) populations (dextramers for NP396, GP33 or NP205). Additionally, the “overall” Vβ/Jβ usage was analyzed by sequencing the dextramer-negative, CD8^+^ T cell population. A detailed description of the procedure was published previously [46,50]. Brief description of the workflow: RNA was extracted using the RNeasy plus microRNA extraction kit (Qiagen). The cDNA synthesis and amplification PCR were performed using the SMARTer RACE cDNA amplification kit (Clontech) and the Advantage 2PCR kit (Clontech). Amplicon size was determined by running an agarose gel and the respective products were indexed with another PCR (Advantage 2PCR kit (Clontech)) and Nextera primer combinations (Illumina). Finally, all sample concentrations were determined and sequencing was performed with a Miseq (Illumina) using V2 chemistry and 150 bp paired-end sequencing.

### 2.6. Sequencing Analysis

Quality control of forward and reverse reads for each individual sample was performed using fastp [51]. Assembling and alignment of reads to TCRβ clonotypes were performed using MiXCR [52] with an “ETE” and otherwise default settings. In order to avoid artificial diversity increases by erroneous sequences, the number of clonotypes was trimmed down by using a 96% cutoff, as described before by others [53]. For this, we sorted the clonotypes in regard to the abundance and cut of T cell receptor clonotypes that appear after 96% of the overall read frequency was reached. This led to the deletion of minor “clonotypes”, detected predominantly with only one or two reads. Warren et al. [53] suggests that the 4% of the repertoire with the lowest abundant clonotypes inflates the diversity due to mostly erroneous sequences from PCR and sequencing. The calculation of the Shannon–Wiener Index was performed with VDJ-tools [54], which normalizes the sequencing reads to the level of the highest sequencing depth (sample with the most reads, extrapolation). Graphical depiction was conducted using R and Prism 7.03 software (GraphPad Software).

### 2.7. Hydrogen Bond Calculations

Peptide MHC crystal structures 1fg2 (GP33/H-2D^b^), 3p4m (NP205/H-2K^b^) and 1jpg (NP396/H-2D^b^) were acquired from the Protein Databank (PDB). PDB crystal structures were processed and visualized using ChimeraX. Briefly, individual peptide–MHC complexes were isolated and trimmed of their β-2 microglobulin and α3 domain. Hydrogen bonds were also calculated with ChimeraX using relaxation constraints of 0.4 angstrom distance and 20-degree angle tolerance. For salt bridges, these constraints were relaxed to 1 angstrom and 20 degrees.

### 2.8. Virus Titer Determination

LCMV titers were determined by plaque assay as performed [46] and described previously [55]. In summary, serial log_10_ dilutions of kidney tissue homogenate were incubated on Vero cells with ∼70% confluence in a 6-well plate. Staining was performed 4 days post infection with neutral red (Sigma-Aldrich, Taufkirchen, Germany); 2 days later, plaques were counted and PFUs per organ were calculated.

### 2.9. Statistics

Descriptive statistics are expressed as means ± standard error of the mean. Depending on the standard deviation, Student’s t tests, Mann–Whitney U tests, analysis of variance (ANOVA), or Kruskal–Wallis tests were performed, using Prism 9.04 software (GraphPad Software).

## 3. Results

### 3.1. NP396-, GP33- and NP205-Specific T Cell Responses Can Be Used to Analyze TCR Repertoires of Differentially Exhausted Epitope-Specific Responses in Chronic LCMV Infection

**Figure 1:**
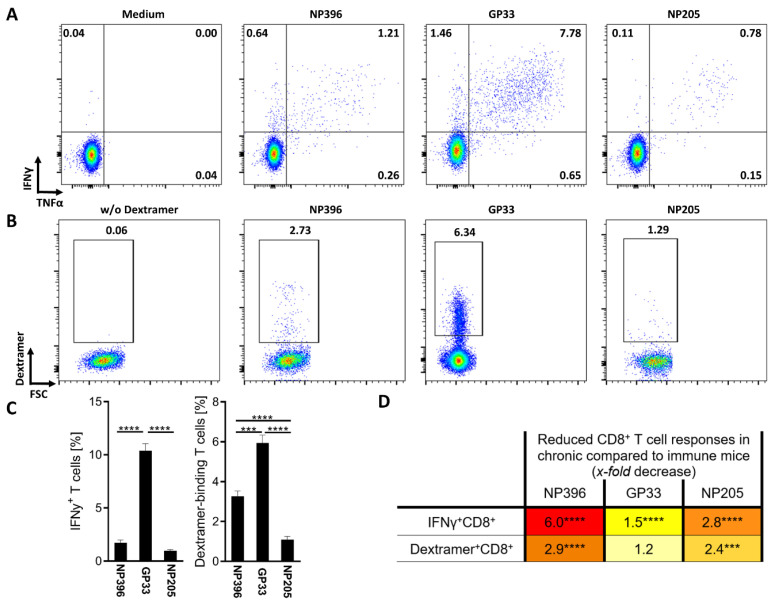
LCMV epitope-specific T cell response alterations were detectable in chronic-LCMV-infected mice. (**A**) Representative plots from one individual mouse showing the frequency of epitope-specific IFNγ^+^CD8^+^CD44^+^ T cells and (**B**) epitope-specific Dextramer^+^CD8^+^CD44^+^ T cells on day 36 post LCMVcl13 infection. (**C**) The frequency of epitope-specific IFNγ^+^CD8^+^CD44^+^ T cells (n = 41–48) and Dextramer^+^CD8^+^CD44^+^ T cells (n = 31–42) is shown. Graphs depict means with standard error of the means (SEM). (**D**) The x-*fold* decrease in T cell responses from LCMV-immune (n = 19–27) to chronically LCMV-infected mice was calculated. Colors represent a range from weak reduction (light yellow) to strong reduction (red). Significance levels were determined by individual statistical comparison between the respective immune and chronic groups and are depicted directly next to the x-*fold* decrease. Significance levels are depicted with *** *p* < 0.001, **** *p* < 0.0001, representative of 9 experiments.

**Figure A2:**
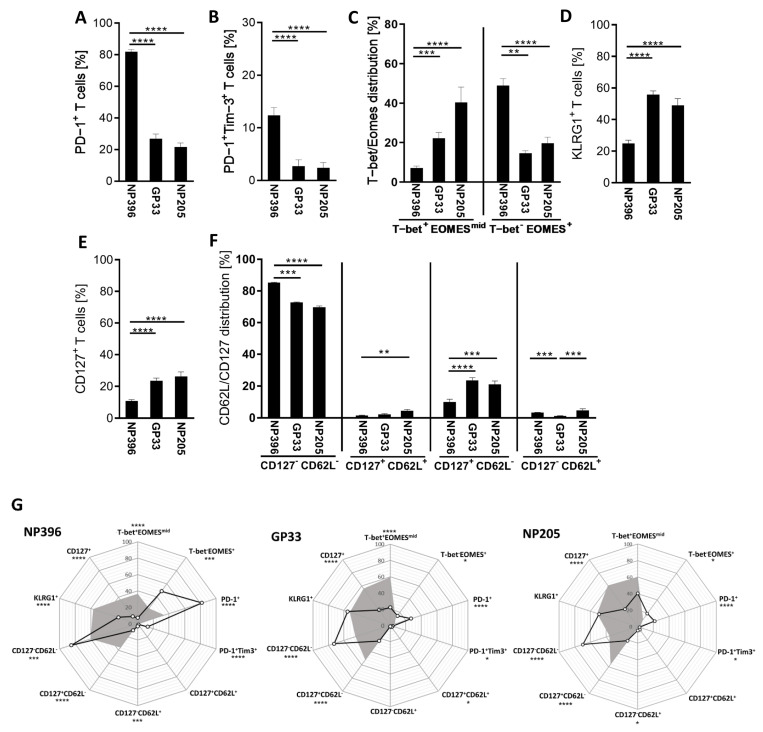
Phenotypical characterization revealed significant differences between the epitope-specific T cells. (**A**–**F**) Flow-cytometric measurement of epitope-specific CD8^+^CD44^+^ T cells gated from Dextramer^+^ populations are shown from chronically LCMV-infected mice. Bar graphs depict means with SEM. (**G**) Radar plots depict phenotypical marker comparisons between LCMVcl13 (black line) and LCMV-immune (grey areas) mice. Stars imply significant differences between LCMCcl13 and LCMV-immune mice. All significance levels are depicted with * *p* < 0.05, ** *p* < 0.01, *** *p* < 0.001, **** *p* < 0.0001, n = 6–26.

The three LCMV-specific T cell responses, NP396, GP33 and NP205, are known to be differentially exhausted in chronically LCMVcl13-infected mice [12]. We could confirm highly significant differences between these three epitope-specific T cell responses, concerning IFNγ^+^ CD8^+^ T cells, dextramer-binding (Dextramer^+^) CD8^+^ T cells and phenotyping of the Dextramer^+^ CD8^+^ T cells (Figure 1A–C and Figure A2).

The highest frequency of IFNγ^+^ and Dextramer^+^ CD8^+^ T cells was detectable in GP33-specific T cells (Figure 1C), while NP396- and NP205-specific responses showed significantly lower frequencies. Comparing NP396- and NP205-specific T cells, NP396-specific T cells showed a significantly higher frequency of Dextramer^+^ CD8^+^ T cells, but similar frequencies of IFNγ^+^ CD8^+^ T cells. In addition to these differences, different phenotypes were detectable in regard to these epitope-specific responses (Figure A2). We could confirm a more exhausted phenotype of NP396-specific CD8^+^ T cells that had significantly higher frequencies of T cells than an exhausted phenotype (PD-1^+^, PD-1^+^Tim3^+^, T-bet^−^Eomes^+^) and significantly less effector T cell characteristics (T-bet^+^Eomes^mid^) compared to the GP33- and NP205-specific responses (Figure A2A–C). GP33-specific T cell responses contained comparable frequencies of exhausted phenotype T cells (PD-1^+^, PD-1^+^Tim3^+^, T-bet^−^Eomes^+^) to the NP205-specific response, but slightly lower frequencies of an effector phenotype (T-bet^+^Eomes^mid^) (Figure A2C). Additionally, most of the epitope-specific T cells were of CD127^−^CD62L^−^ phenotype (effector and exhausted cells). Nevertheless, CD127^−^ and long-lived CD127^+^ T cells showed significant phenotypical differences between the three investigated epitope-specific responses (Figure A2E,F). In line with the other phenotypical characteristics, the NP396-specific T cell responses revealed the lowest frequency of long-lived T cells, while the GP33- and NP205-specific T cell responses were similar.

**Figure A3:**
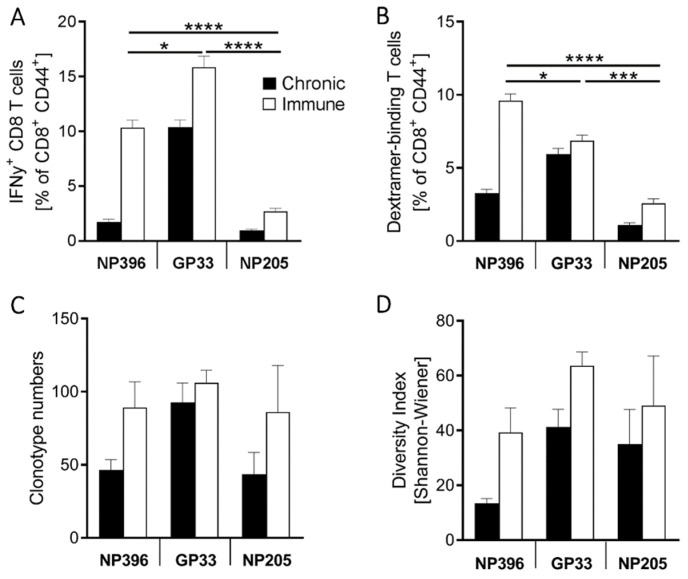
Epitope-specific alterations were detectable in LCMV-specific T cell responses. Epitope-specific T cells were analyzed at day 36 post LCMVl13 (chronic) and LCMV (immune) infection. (**A**) Frequency of epitope-specific IFNγ^+^CD8^+^CD44^+^ T cell responses, (**B**) frequency of Dextramer^+^CD8^+^CD44^+^ T cells (n = 33–44) and (**C**) number of epitope-specific clonotypes are shown and (**D**) a diversity index of epitope-specific clonotypes is depicted (n = 6–18). Graphs depict means with standard error of the means (SEM), significance levels comparing the T cells in immune mice (white bars) are given, with * *p* < 0.05, *** *p* < 0.001, **** *p* < 0.0001.

To further confirm the exhaustion levels of the epitope-specific T cell responses, we compared the responses in chronically infected mice to those of acute resolved LCMV (named “immune”) mice, detecting overall decreased responses, but at an individual epitope-specific degree (Figure 1D). As previously shown, NP396^+^-specific CD8^+^ T cells showed the most severe, highly significant exhaustion [46]. The subdominant NP205-specific response also displayed a significant, 2.8-*fold* decrease in the frequency of IFNγ^+^ T cells, while the difference in GP33-specific responses was less pronounced between chronically LCMV-infected and LCMV-immune mice. The phenotype of these responses is also individual and differs between the epitope-specific T cell responses, although all showed a major shift in phenotype from immune to chronic mice (Figure A3A,B).

### 3.2. NP396-, GP33- and NP205-Specific T Cell Responses Displayed Differences in the TCR Repertoire in Chronic LCMV Infection

**Figure 2:**
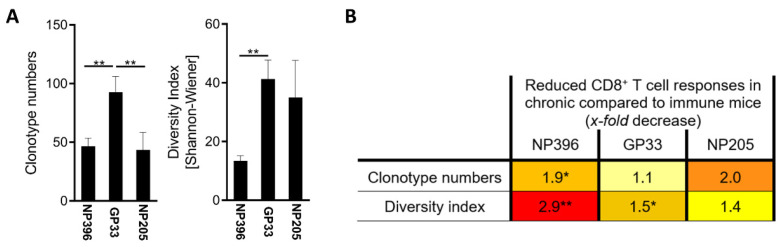
Epitope-specific differences in exhaustion translated to a different TCR repertoire. (**A**) The number of epitope-specific clonotypes from all Dextramer^+^CD8^+^CD44^+^ T cells and the diversity index of epitope-specific clonotypes is depicted day 36 post LCMVcl13 infection. Graphs depict means with standard error of the means (SEM). (**B**) The x-*fold* decreases in the T cell responses from LCMV-immune to chronically LCMV-infected mice were calculated. Colors represent the range from weak reduction (light yellow) to strong reduction (red). Significance levels were determined by individual statistical comparison between the respective immune and chronic groups and are depicted directly next to the x-*fold* decrease. Significance levels are depicted with * *p* < 0.05, ** *p* < 0.01, n = 7–18, representative of 3–5 experiments.

The number of clonotypes and diversity of the TCR repertoire were determined by next-generation sequencing of Dextramer^+^ T cells. A significantly higher number of GP33-specific clonotypes was detected compared to NP396- and NP205-specific T cells (Figure 2A), which is consistent with the analysis of IFNγ^+^ and Dextramer^+^ CD8^+^ T cells (Figure 1C). No differences were observed between the number of NP396- and NP205-specific clonotypes (Figure 2A). A higher GP33-specific clonotype number translated into significantly higher diversity compared to NP396-specific responses, but no differences were seen in NP205-specific responses. The diversity in NP396-specific T cell responses was lower than in NP205-specific responses. Comparing the constraints of the TCR repertoire between immune and chronic mice, we found significant decreases in clonotype number and diversity (Figure 2B and Figure A3C,D), with most prominent changes being revealed again in the response to NP396, followed by NP205-specific TCR responses.

These results indicate that the TCR repertoire of the investigated LCMV epitope-specific T cell responses evolves with the stage of exhaustion during chronic infection. The immune mice were further used as another benchmark to compare TCR-related differences between those epitopes.

### 3.3. Distinct Vβ/Jβ Patterns Appeared in Individual Epitope-Specific TCR Repertoires

Altered clonotype number and diversity between the epitope-specific T cell responses in chronically infected mice (Figure 2A) indicated that the TCR repertoire might be altered. Therefore, we analyzed Vβ- and Jβ-chain usage, as well as the length of the complementary determining region 3 (CDR3) of the respective TCR repertoires. Additionally, we compared those results with T cell responses in LCMV-immune mice to detect exhaustion associated shifts.

**Figure 3:**
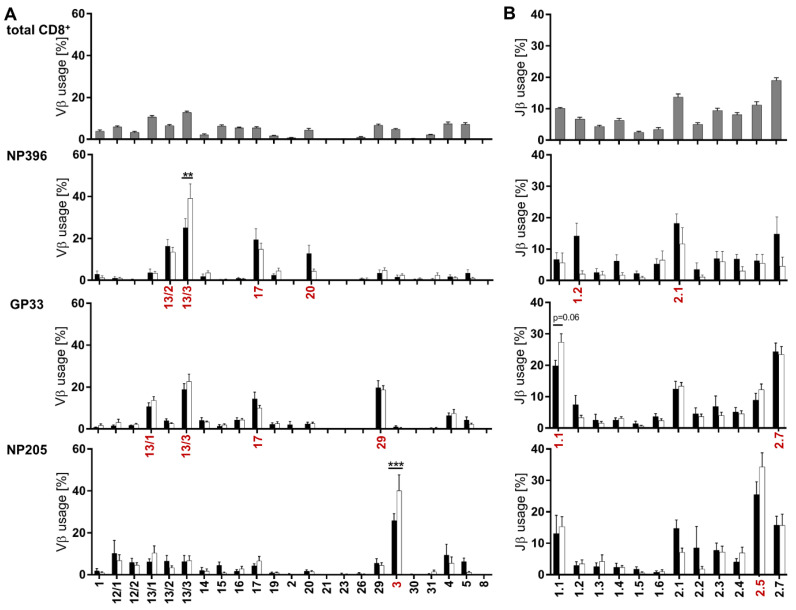
Vβ and Jβ usages were distinctly different between the analyzed epitope-specific responses. (**A**) Vβ-chain and (**B**) Jβ-chain usage is shown for TCRβ clonotypes of all CD8^+^ T cells (grey bars; all mice) and the three epitope-specific CD8^+^CD44^+^ T cell responses against NP396, GP33 and NP205 in chronically LCMV-infected (black bars) and immune mice (white bars). Graphs depict means with SEM, red labels indicate predominant usage within the respective epitope-specific response. Significance levels were depicted with ** *p* < 0.01 and *** *p* < 0.001, n = 6–18, representative of 3–5 experiments.

Analysis of the Vβ- and Jβ-chain usage showed that all three epitope-specific T cell responses had a prominent increase in distinct Vβ- and Jβ-chain usages in comparison to the overall Vβ/Jβ usage of CD8^+^ T cells (Figure 3). NP396- and GP33-specific T cell responses predominately used four Vβ-chains and two Jβ-chains, whereas NP205-specific responses were focused on one Vβ- (Vβ3) and one Jβ-chain (Jβ2.5) in chronically infected mice (Figure 3, marked in red). Interestingly, the previously shown significant differences between chronic and immune mice (Figure 2B) induced only minor shifts in the Vβ- and Jβ-chain usage, despite two significant shifts (NP396 Vβ-13/3 and NP205 Vβ-3) (Figure 3A).

**Figure 4:**
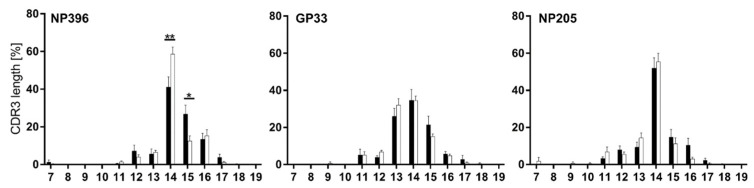
TCRβ CDR3 length revealed minimally different distributions between the epitope-specific responses. CDR3 length distribution from all TCRβ clonotypes is depicted for NP396-, GP33- and NP205-specific CD8^+^CD44^+^ T cells in chronically LCMV-infected (black bars) and immune mice (white bars). Graphs depict means with SEM, significance levels are shown with * *p* < 0.05 and ** *p* < 0.01, n = 6–18, representative of 3–5 experiments.

CDR3 length measurements revealed that all three epitope-specific responses favored 14 amino acids (aa) (Figure 4). This preference followed an approximately normal distribution for GP33, but was more pronounced for NP396 and especially for NP205.

In line with data shown in Figure 3, only minor changes appeared between TCR usage of chronic and immune mice, with significant shifts only in the NP396-specific T cell response (CDR3 length 14aa and 15aa) (Figure 4).

Overall, quantitative comparison indicated that NP396- and GP33-specific responses used more different Vβ- and Jβ-chains and CDR3 length compared to NP205-specific responses.

### 3.4. The TCR Repertoires to NP396 and GP33 Were Highly Private, Whereas NP205-Responses Revealed a Public TCR Repertoire

The Vβ- and Jβ-chain usage depends on the MHC composition of the host and is therefore relatively stable in the used genetically identical C57Bl/6 mice. However, on the aa level of the CDR3, public sequences, or closely related sequences (following a motif), are rarely seen among the highly abundant clonotypes, e.g., NP396 [46]. To further investigate whether this observation is also true for LCMV GP33- and NP205-specific responses, we analyzed the CDR3 aa sequences. Within the dominant NP396- and GP33-specific usages, no public sequences or repetitive motifs were detectable, revealing so-called private TCR repertoires.

**Figure 5:**
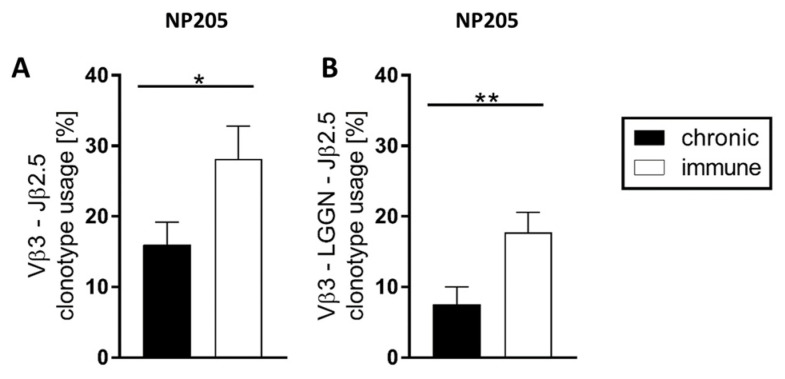
NP205-specific T cell responses showed a public Vβ3-‘LGGN’-Jβ2.5 pattern for clonotype selection. The relative frequency of (**A**) the most dominant Vβ3-Jβ2.5 combination and (**B**) the specific ‘LGGN’-clonotype in response to NP205 is depicted in chronically LCMV-infected (black bars) and immune mice (white bars) with means and SEM, significance levels are * *p* < 0.05, ** *p* < 0.01, n = 7–11, representative of 3 experiments.

In contrast, NP205-specific T cell responses were very focused on Vβ3 and Jβ2.5 (Figure 3), and these two dominant chains were paired for a relatively high frequency of 16% in chronic and 28% in immune mice (Figure 5A). In these Vβ3/Jβ2.5-clonotypes, a motif was detectable, using a four aa connection in the center of the CDR3, which is L-G-G-N (Leucine, Glycine, Glycine, Asparagine) or closely related to this (single aa changes). The relative frequency of this clonotype within the TCR repertoire is significantly reduced in chronic compared to immune mice (Figure 5B). Interestingly, most of the ‘LGGN’-clonotype carrying mice showed multiple nucleic acid sequences leading to the ‘LGGN’-clonotypes, indicating multiple selections of this clonotype in the thymus.

Concluding, NP396- and GP33-specific responses were found to be private, with neither a public clonotype nor a public motif. Therefore, the very profound dependence of the NP205-specific responses on one public motif was a unique feature. This separated NP205-specific from NP396- and GP33-specific T cell responses.

After finding these individual epitope-specific T cell responses, we were interested in knowing whether these epitope-specific responses influence and interact with each other. Therefore, we compared different epitope-specific T cell responses isolated from individual mice (Table A1). We could not find any indications of dependencies (e.g., substitution, suppression) between those epitope-specific responses searching for those correlations of and across many factors between epitopes.

Our data show that the exhaustion of LCMV-specific T cell responses is epitope-specifically independent and different in strength, phenotype and TCR repertoire in chronic infection.

### 3.5. Checkpoint Inhibitor Treatment Shaped Predominantly the NP396- and NP205-Specific CD8^+^ TCR Repertoire, but Had Only Minor Effects on the GP33-Specific One

**Figure 6:**
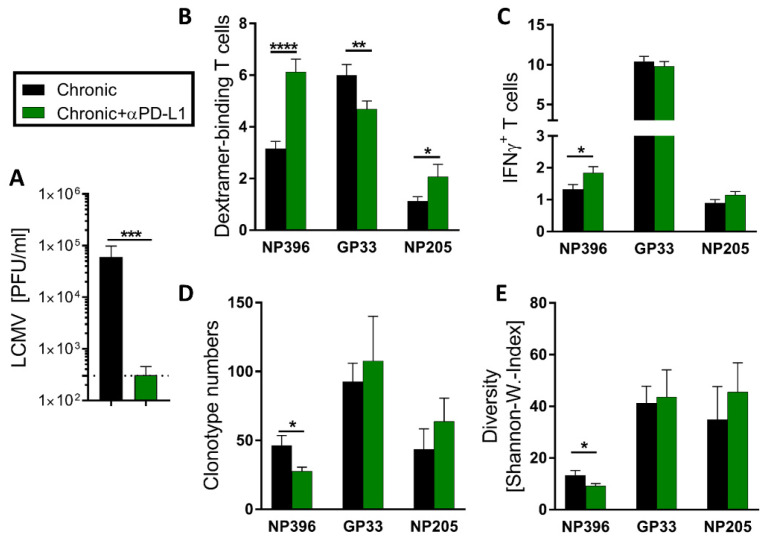
Individual shaping of the epitope-specific T cell responses by checkpoint inhibitor αPD-L1 treatment. (**A**) Viral titers were determined in the kidney at day 36 after LCMVcl13 infection (n = 23–45). (**B**,**C**) LCMV NP396-, GP33- and NP205-specific CD8^+^CD44^+^ T cell responses were measured by Dextramer and IFNγ staining (n = 33–45). (**D**) The number of NP396-, GP33- and NP205-specific TCRβ clonotypes and (**E**) the resulting diversity (calculated of the repertoire with the Shannon–Wiener Index) was determined by sequencing (n = 6–18). Graph depicts means with SEM, significance levels with * *p* < 0.05, ** *p* < 0.01, *** *p* < 0.001, **** *p* < 0.0001, representative of 3–5 experiments.

Anti-PD-L1 treatment in chronic-LCMV-infected mice restores exhausted virus-specific T cell responses and supports viral clearance (Figure 6A) [12,46]. Knowing from our data that LCMV epitope-specific T cell responses are differently exhausted in chronic LCMV infection, we were interested whether these differences also transfer into different sensitivity to treatment with ICI. We have previously reported profound effects of αPD-L1 on the response against NP396 [46]. Extending this analysis, we confirmed that the effect of αPD-L1 on NP396-specific responses resulted in significantly increased frequencies of Dextramer^+^ CD8^+^ T cells and IFNγ^+^ NP396-specific CD8^+^ T cells, as well as reduced clonotype number and diversity (Figure 6B–E). The response to NP205 was comparable to NP396-specific T cells in regard to a significantly increased frequency of Dextramer^+^ T cells and increased frequency of IFNγ^+^ NP205-specific CD8^+^ T cells (Figure 6B,C). However, whereas the clonotype number and the diversity of NP396-specific T cells were significantly reduced in αPD-L1-treated mice, the clonotype number and diversity of NP205-specific T cells were increased in tendencies compared to chronically LCMV-infected mice (Figure 6D,E). The frequency of GP33-Dextramer^+^ T cells decreased upon αPD-L1 therapy (Figure 6B), which could be a relative result from other responses increasing, rather than GP33-specific frequencies decreasing. Other than that, the αPD-L1 treatment showed hardly any effect on the GP33-specific CD8^+^ T cells (Figure 6C–E).

In contrast to NP396-specific responses, TCR repertoire sequencing of GP33- and NP205-specific T cells could not show significant modulations in TCR clonotype number (Figure 6D) or diversity (Figure 6E) from αPD-L1 treatment. However, NP205-specific responses displayed increased Dextramer^+^ T cell frequencies and clear tendencies towards broader TCR repertoires after treatment.

Our data showed that the responsiveness to ICI therapy was pronounced to NP396-specific T cell responses, whereas the responses against GP33 and NP205 were rather unaffected, revealing heterogeneous effects of ICI therapy on the virus epitope-specific level.

### 3.6. Crystallographic Structures of Peptide–MHC Complexes Indicated Differences in Epitope-Specific Antigen Presentation to CD8^+^ T Cells

**Figure 7:**
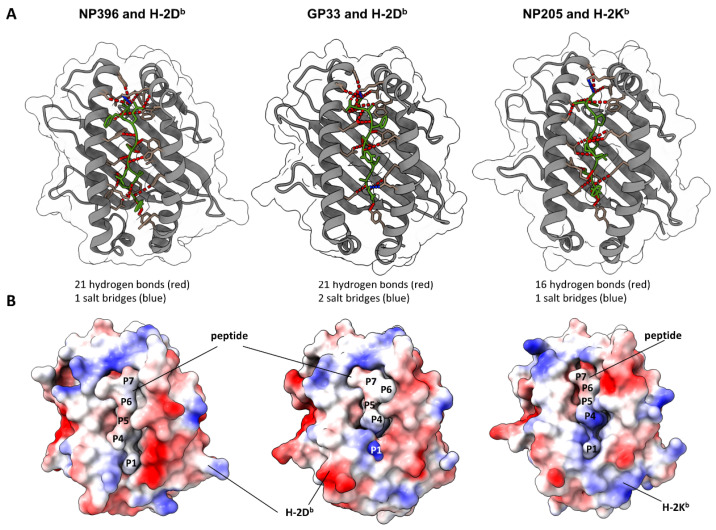
Modeling of peptide–MHC complexes revealed different binding properties and electrostatic potential. (**A**) UCSF ChimeraX-based visualization of X-ray crystallographic protein structures. MHCs H-2D^b^ (NP396 and GP33) and H-2K^b^ (NP205) are depicted in grey, respective peptides in green, anchor molecules in light brown. Hydrogen bonds are depicted in red, salt bridges in blue. (**B**) Electrostatic potential is depicted on a bubble structure by UCSF ChimeraX. Visualizing the peptide centrally within the binding groove of the MHC molecule. Red is negatively charged, blue is positive and white is neutral.

Aside from different T cell clonotypes, epitope-specific T cell responses might also be shaped by different peptide presentations. To assess differences in this regard and appreciate the antigen presentation as a major factor for the shown epitope-specific differences, we obtained crystal structure models of the peptide–MHC complexes for our three analyzed peptides from the Protein Databank. These structures were used as input to compute peptide-MHC bonds and the electrostatic potential over the complex surface using UCSF ChimeraX. The calculation predicted 21 hydrogen bonds for NP396, 21 for GP33 and 16 for NP205 peptide binding to the MHC (Figure 7A). Additionally, one, two and one salt bridge(s) were predicted, respectively. Electrostatic coloring showed differences from a rather neutral and exposed NP396 peptide to a more hidden peptide with a positively charged P1 on GP33 (dark blue, lower part of the peptide, Lysine), and a more positively charged P4 on the NP205 peptide (Figure 7B). These different patterns of interaction reflect local rearrangements within the MHC-binding cleft which can affect peptide–MHC stability and presentation to the T cells. In addition, the different patterns observed for the peptide–MHC surfaces are consistent with different T cell populations being triggered by each one of these peptides. These findings might help to understand the detected differences in the epitope-specific CD8^+^ T cell responses.

## 4. Discussion

To our knowledge, this is one of the first studies comparing multiple epitope-specific CD8^+^ T cell responses within one chronic, virus-specific response in depth in regard to TCR repertoire, peptide presentation and responsiveness to ICI therapy. Many studies have investigated T cell responses in chronic viral infections focusing either on “the immune response” by combining individual epitope-specific responses or by using one epitope-specific response, extrapolating this knowledge to all virus epitope-specific responses (the overall response) [56,57,58]. Recently, we and others started to analyze and describe the vast variety that already appears on the level of epitope-specific responses in chronic infections [59,60,61,62]. In this study, we demonstrated that the three LCMV epitope-specific T cell responses (NP396, GP33, NP205), which are known to be individual in the strength of the induced response, loss of functionality and phenotype within a chronic immune response, revealed major differences in TCR repertoire and ICI treatment responsiveness.

For the NP396-specific response, we confirmed and extended the previously reported, severely exhausted phenotype [10,46,63], and found diminished clonotype number and diversity indices [46], accompanied by an oligoclonal TCR repertoire. The overall response revealed a high sensitivity to ICI treatment which resulted in even more pronounced oligoclonality in the TCR repertoire. The GP33-specific response dominated the LCMV-specific T cell responses and displayed significantly less exhaustion compared to NP396- as well as NP205-specific responses. Overall, all acquired data indicated that GP33-specific responses remained less affected during chronic LCMV infection, even under ICI therapy. The NP205-specific response showed similarities to the NP396 responses. In contrast to NP396 and GP33, the NP205-specific response showed a greater use of one public Vβ3/Jβ2.5-clonotype motif. Why this occurred in the NP205-specific response is still unknown. There is no indication that this is a feature of the H-2K^b^, preferring Vβ3 binding. The fact that this ‘LGGN’-clonotype has been described not only for NP205-specific responses in LCMV-immune C57Bl/6 mice [44], but also in the p277 peptide (heat shock protein) [64] and in 27 out of 28 naïve C57Bl/6 mice [65], indicates its ubiquities presence. Therefore, it is likely that this clonotype is close to universal in C57Bl/6 mice, able to expand in nearly all mice in response to NP205 and probably related to a thymus-dependent selection process preferring this clonotype.

Besides the individual comparison of the three epitope-specific responses, we examined interactions between multiple epitope-specific responses by analyzing them within the same mouse. In correlation analysis, we found no dependencies, further strengthening our understanding of individual, epitope-specific T cell responses. Alternatively, one could suggest that the number of mice used in this study was too low to find significant dependencies. However, none of the analyzed interaction combinations were close to a significant correlation, while many significant differences appeared in other analyses. The absence of such dependencies is in line with previous results, showing intra-epitope competition for T cell responses [66,67], rather than across different epitopes, which is especially true in LCMV [68,69].

ICI therapy has been implemented as a very successful treatment in the clinics against cancer, and is also being evaluated for chronically hepatitis-B-virus-infected patients [70], although not all patients respond to it and immune responses are quite heterogeneous. It is known that this heterogeneity can originate, for example, from different levels of PD-1 expression on immune cells or mutations in the *pdcd-1* gene [71]. However, little is known about whether epitope-specific differences also occur.

Anti-PD-L1 therapy in chronically LCMV-infected mice is effective for viral clearance [12,46]. We previously reported a profound effect on the TCR repertoire against NP396 [46] and showed in this study that the ICI therapy modulates the TCR repertories responding to GP33 and NP205 significantly less. Additionally, the effects on the number of responding T cells and function were lower, revealing overall epitope-specific differences in ICI responsiveness, which is in line with previous results [12]. This could be a consequence from less exhaustion of these responses or a dominant phenotype of T cells in the response that are not affected by ICI therapy (e.g., TCF1^−^, [15]). In contrast to suggestions that less-exhausted T cells profit most from ICI therapy, we found the most prominent effect in the most-exhausted, NP396-specific response in a model system that undeniably profits from the treatment. Nevertheless, both results are in line with each other, when hypothesizing that the few remaining “less exhausted” T cells within NP396-specific responses are the origin of the detected effects. The low number of these cells could also explain the massive oligoclonality that is detected after ICI therapy. It has to be mentioned that frequency of cells was shown, since we focus on the differences between the epitope-specific responses. Due to the overall increase in cells, the GP33-specific T cell frequency is significantly reduced, but the absolute cell number is not significantly decreased after αPD-L1 treatment. Nevertheless, the shown frequency strengthens the point that GP33-specific responses are severely less affected than NP396-specific ones. Although it is currently impossible to target specific responses with ICI therapy, we would speculate on the basis of our data that restoring very exhausted epitope-specific responses, in order to restore broader epitope coverage, is more valuable than pushing less-exhausted ones. In the end, the question remains whether ICI-induced restoration actually saves T cells from exhaustion rather than boosting the subsequently responding T cells [72]. Dahling et al. have suggested that the response is “unleashed” by ICI therapy [73]. This is in line with the published data indicating that the phenotype of these restored T cells might be more exhausted [74].

Besides the T cells themselves, the importance of antigen presentation is also indicated by our results. NP396- and GP33-specific responses showed very private TCR repertoires between mice, but developed consistently in their individual phenotype throughout a chronic LCMV infection in all C57Bl/6 mice, indicating an unknown force guiding this development. Since each mouse used different TCR clonotypes, a TCR-independent factor has to be a major regulator for this. A likely explanation is that different antigen presentations of both peptides, originating from different binding affinities or stabilities of the respective peptide to the MHC H-2D^b^, allow for more or less presentation, T cell activation and functional T cell avidity. Our depicted modeling of the peptide–MHC complexes supports this concept of different antigen presentations, revealing different amounts of hydrogen bonds and distinctive electrostatic surfaces. While anchor amino acids (P2 and P9) appear similar between both complexes [75], the most differences are visible in the amino acids P5, P6 and P7 [76]. In this context, it has also been shown that NP396- H-2D^b^ binding is much more profound than GP33-H-2D^b^ binding [42], possibly supporting more TCR binding and faster exhaustion of the NP396-specific response. Importantly, GP33 can be presented by H-2D^b^ and H-2K^b^ [77], which can be hypothesized to support the observed more diverse TCR response. These data indicate that epitope-specific alterations may partially originate from the MHC side, indicating that the antigen presentation is important to further explain epitope-specific differences. In regard to the NP205 motif, we hypothesize that it originates from the presence of the ubiquitous LGGN-clonotype prior to infection. Consequently, no such ubiquitous clonotype responds in high abundance to NP396 or GP33; therefore, no dominant motifs are detectable in these responses between different mice. In conclusion, the reason for the different development of epitope-specific responses is beyond the scope of this study. We think that the differences in antigen presentation are a major reason for our reported differences in exhaustion. Furthermore, we hypothesize that differences such as TCR changes and ICI responsiveness rely on the exhaustion status of the individual responses and may change over time. Indications for this can be drawn from a comparable LCMV mouse model where CD4^+^ T cells are depleted in order to generate a deeper exhaustion state. Under these slightly more artificial conditions, GP33-specific TCR repertoires are also significantly shifted [47].

The strength of this study is that we have revealed three very different epitope-specific responses with different exhaustion statuses (effect of exhaustion on the TCR repertoire and ICI treatment responsiveness) by analyzing nearly 50 mice, with flow cytometry as well as deep-sequencing techniques. We analyzed different epitope-specific responses within individual mice in order to systemically compare these responses in depth and find dependencies, interactions or substitutions. Some recent analyses of other TCRs have shown how phenotypically heterogeneous epitope-specific T cell responses can be [41,78], although they have identical TCR-carrying T cells [31,40,62]. Although these transgenic models are slightly more artificial with high numbers of GP33-specific P14 T cells, it nevertheless shows the restrictions of TCR repertoire and antigen presentation analysis, and that other important factors are yet to be found. A specific limitation of this study is that only TCRβ was determined. While being widely accepted as the main driver for TCR repertoire characteristics, the TCRα [79] and dual TCRα T cells (although mostly in CD4^+^ T cells) [80] should have an impact. It is also worth noting that we could not combine phenotypical and TCR data on a single-cell level, which will be an interesting topic of future work.

## 5. Conclusions

In this study, we showed that epitope-specific T cell responses are individual and independent. Therefore, T cells should be analyzed on an individual epitope level, rather than summarizing all to “one immune response”. Epitope-specific differences might help explain heterogeneity in immune responses from patients with chronic viral infections and cancer and in terms of responsiveness to ICI therapy. Our findings might open additional paths to generating new therapeutic options.

## Data Availability

Data will be made available upon publication on a local server RepoMed (mhh-publikationsserver.gbv.de).

## Appendix A

**Table A1:** All mice that were used for TCR repertoire analysis are shown, with respective, measured epitope-specific TCR repertoires.

		NP396				GP33				NP205			
Mouse	Treatment	Cells (Input)	Reads	Clonotypes	Diversity	Cells (Input)	Reads	Clonotypes	Diversity	Cells (Input)	Reads	Clonotypes	Diversity
1	chronic					9000	15,551	72	24.0				
2	chronic					9000	15,584	52	28.1	830	103,183	21	11.2
3	chronic					11,000	28,628	62	23.8				
4	chronic	9800	28,242	30	10.9	11,800	24,887	151	78.4				
5	chronic	1840	18,410	38	21.3	12,100	28,764	88	41.5				
6	chronic	2540	19,488	16	9.1	8800	18,303	70	34.9				
7	chronic	10,100	30,510	37	11.2	12,200	35,831	100	40.2				
8	chronic	8400	27,096	33	11.0								
9	chronic	12,300	30,587	54	9.2								
10	chronic	10,000	20,962	30	14.9								
11	chronic	11,300	20,164	33	15.7								
12	chronic	10,000	20,152	26	5.1								
13	chronic	8800	21,742	35	9.9								
14	chronic	6200	28,333	47	8.9								
15	chronic	6800	26,948	51	23.0								
16	chronic	6100	21,298	147	36.2								
17	chronic	5900	27,859	53	12.8								
18	chronic	10,400	3675	49	5.7								
19	chronic	9800	17,082	86	8.2								
20	chronic	10,000	24,239	54	17.6								
21	chronic					11,100	41,995	182	77.8	4022	9511	23	19.2
22	chronic					15,100	37,435	59	27.2	1441	12,797	22	18.9
23	chronic					10,800	8893	91	37.3	2540	14,480	124	103.5
24	chronic									5547	37,082	60	46.4
25	chronic									2190	34,981	45	39.6
26	chronic									11,900	28,049	10	6.2
27	chronic	6500	25,837	53	12.8								
28	chr.+αPD-L1	9600	27,130	33	10.5	8000	25,475	102	44.2				
29	chr.+αPD-L1	10,000	28,383	12	5.5	9000	21,762	134	63.4				
30	chr.+αPD-L1	9460	32,553	29	7.9	14,200	31,020	63	23.6				
31	chr.+αPD-L1	11,300	27,835	29	10.8	9400	28,884	116	40.0				
32	chr.+αPD-L1	11,000	16,900	35	14.6								
33	chr.+αPD-L1	11,000	17,275	29	10.4								
34	chr.+αPD-L1	10,000	12,981	8	5.2								
35	chr.+αPD-L1	10,000	17,407	24	13.3								
36	chr.+αPD-L1	13,000	15,704	36	13.8								
37	chr.+αPD-L1	12,000	20,618	28	8.7								
38	chr.+αPD-L1					10,200	40,833	276	92.4	2972	14,982	77	56.4
39	chr.+αPD-L1					10,200	37,557	10	7.3	2861	13,683	74	52.1
40	chr.+αPD-L1					11,100	42,348	54	34.4	2536	8823	105	63.7
41	chr.+αPD-L1	10,000	13,757	20	8.6					5711	40,250	101	77.8
42	chr.+αPD-L1									3722	46,224	12	10.4
43	chr.+αPD-L1	14,700	13,123	10	4.6					13,500	29,309	14	12.9
44	immune					13,600	36,281	156	89.8	5088	20,027	124	71.2
45	immune	11,500	17,831	33	17.0					7865	38,368	15	13.6
46	immune	13,600	21,382	89	37.1					13,600	32,486	86	48.3
47	immune	13,600	17,748	44	15.1					13,400	40,100	23	16.2
48	immune	15,500	23,349	141	70.9								
49	immune	10,000	22,435	124	56.8								
50	immune	8900	20,648	104	39.0								
51	immune					10,000	19,338	97	45.6	6003	16,6205	177	67.4
52	immune					10,000	23,712	97	60.9	6068	35,970	9	8.2
53	immune					10,000	32,581	108	60.0	5709	216,712	362	215.7
54	immune					9500	24,943	76	48.1	11,020	64,975	9	8.3
55	immune					10,000	22,220	101	65.4	5140	168,059	73	44.6
56	immune					10,000	24,534	125	76.6	15,393	108,220	23	20.3
57	immune					10,000	20,314	88	62.0	3408	135,541	45	25.7
